# Navigated functional alignment total knee arthroplasty achieves reliable, reproducible and accurate results with high patient satisfaction

**DOI:** 10.1007/s00167-023-07327-w

**Published:** 2023-03-14

**Authors:** Kaushik Hazratwala, Conor Gouk, Matthew P. R. Wilkinson, William B. O’Callaghan

**Affiliations:** 1 The Orthopaedic Research Institute of Queensland (ORIQL), 7 Turner Street, Pimlico, Townsville, QLD 4812 Australia; 2 Mater Health Services North Queensland Ltd, 21-37 Fulham Road, Pimlico, Townsville, QLD 4812 Australia; 3 Townsville University Hospital, 100 Angus Smith Drive, Douglas, Townsville, QLD 4814 Australia; 4 Cairn Base Hospital, 165 Esplanade, Cairns, QLD 4870 Australia; 5 James Cook University, 1 James Cook Drive, Townsville, QLD 4811 Australia; 6 University of Tasmania, Churchill Avenue, Hobart, TAS 7005 Australia

**Keywords:** Functional alignment, Kinematic alignment, Mechanical alignment, Total knee arthroplasty, Total knee replacement

## Abstract

**Purpose:**

The decision on which technique to perform a total knee arthroplasty (TKA) has become more complicated over the last decade. Perceived limitations of mechanical alignment (MA) and kinematic alignment (KA) have led to the development of the functional alignment (FA) philosophy. This study aims to report the 2-year results of an initial patient cohort in terms of revision rate, PROMs and complications for Computer Aided Surgery (CAS) Navigated FA TKA.

**Methods:**

This paper reports a single surgeon’s outcomes of 165 consecutive CAS FA TKAs. The final follow-up was 24 months. Pre-operative and post-operative patient-reported outcome measures, WOMAC and KSS, and intra-operative CAS data, including alignment, kinematic curves, and gaps, are reported. Stress kinematic curves were analysed for correlation with CAS final alignment and CAS final alignment with radiographic long-leg alignment. Pre- and post-operative CPAK and knee phenotypes were recorded. Three different types of prostheses from two manufacturers were used, and outcomes were compared. Soft tissue releases, revision and complication data are also reported.

**Results:**

Mean pre-operative WOMAC was 48.8 and 1.2 at the time of the final follow-up. KSS was 48.8 and 93.7, respectively. Pre- and post-operative range of motion was 118.6° and 120.1°, respectively. Pre-operative and final kinematic curve prediction had an accuracy of 91.8%. CAS data pre-operative stress alignment and final alignment strongly correlate in extension and flexion, *r* = 0.926 and 0.856, *p* < 0.001. No statistical outcome difference was detected between the types of prostheses. 14.5% of patients required soft tissue release, with the lateral release (50%) and posterior capsule (29%) being the most common.

**Conclusion:**

CAS FA TKA in this cohort proved to be a predictable, reliable, and reproducible technique with acceptable short-term revision rates and high PROMs. FA can account for extremes in individual patient bony morphology and achieve desired gap and kinematic targets with soft tissue releases required in only 14.5% of patients.

**Level of evidence:**

IV (retrospective case series review).

**Supplementary Information:**

The online version contains supplementary material available at 10.1007/s00167-023-07327-w.

## Introduction

The decision on which technique to perform a total knee arthroplasty (TKA) has become increasingly complicated. Despite technological improvements, reported dissatisfaction rates remain as high as 20% [14, 17, 19]. To address this, surgeons have begun to explore alternate alignment philosophies.

Contemporary TKA surgical techniques are typically derived from one of two alignment philosophies: mechanical alignment (MA) or kinematic alignment (KA) [6, 9, 14, 18, 19, 22, 23, 25]. To improve patient outcomes beyond MA and KA, another alignment philosophy has been developed: functional alignment (FA) [16]. FA differs from MA and KA in that rather than adhering to strict alignment principles that may result in profound intra-operative challenges in balancing a TKA, priority is instead afforded to achieving knee kinematics that most closely approximates the natural pre-arthritic knee whilst simultaneously preserving the soft tissues with minimal releases and positioning the prosthesis in such a way that restores joint line height, obliquity and pre-arthritic constitutional alignment; aiming to implant the components with minimal compromise of the soft-tissue envelope by restoring the plane and obliquity of the non-arthritic joint [2, 24].

Current literature only reports on FA using robotics, but not CAS navigation. The purpose of this study is to report the 2-year results of the senior author’s consecutive patient cohort in terms of alignment, balance, PROMs, revision rate, and complications specifically for CAS Navigated FA TKA (Supplement 1). The hypothesis was that favourable navigated FA results can be achieved without the aid of robotics at the two-year post-operative point. It is essential to establish the viability of CAS Navigated FA as access to robotic platforms continues to have several obstructions and limitations that if left as the sole option, would prevent surgeons from utilising FA [15].

## Materials and methods

Functional alignment (FA) is a term describing an alignment philosophy that has emerged to address the various limitations incurred by MA and KA techniques [16, 21]. The algorithm for this iteration of FA is contained within Supplement 2: explanation of the FA Rationale and Surgical Technique or see O’Callaghan et al. [20].

### Patient cohort

This cohort study used prospectively collected data by a single surgeon at a single site between September 2016 and February 2020 on 140 consecutive patients. Selection criteria included all adult male and female patients with a primary diagnosis of osteoarthritis for which a navigated primary single or bilateral total knee arthroplasty was performed. UKR and revision TKA patients were excluded. Demographic data were collected on all patients. All patients were evaluated for the Western Ontario and McMaster Universities (WOMAC) and Knee Society Score (KSS) preoperatively and post-operatively at one and two years. Implant-dependent sub-analysis of PROMs was performed: Supplement 3. Revisions and implant-related failures were also reported.

## Demographic data

140 subjects’ data were collected, amounting to 165 knees. Mean age was 65.07 years (SD ± 8.25, Minimum 43 years, Maximum 85 years). 77 subjects were female (55%), and 63 were male (45%). Of the knees, 89 were left (53%), 76 were right (46%) with 25 subjects having bilateral procedures (18%). The DePuy Attune prosthesis was used in 139 procedures (84%)—cruciate retaining (CR) in 52 (31%), and rotating platform in 87 (52%)—and Stryker Triathlon CR in the remaining 26 knee (16%).

### Radiographic evaluation

All patients in this series underwent pre-operative standard anterior to posterior (AP) and lateral radiographs, as well as either standing radiographs or EOS scans for alignment. Postoperative long-leg standing radiographs were taken at 6 weeks postoperatively. Additionally, standard AP and lateral radiographs were performed at 6, 12 and 24 months. Alignment classification groups of Hirschmann’s functional knee phenotypes and Coronal Alignment of the Knee (CPAK), were recorded [12, 13, 18].

**Fig. 1:**
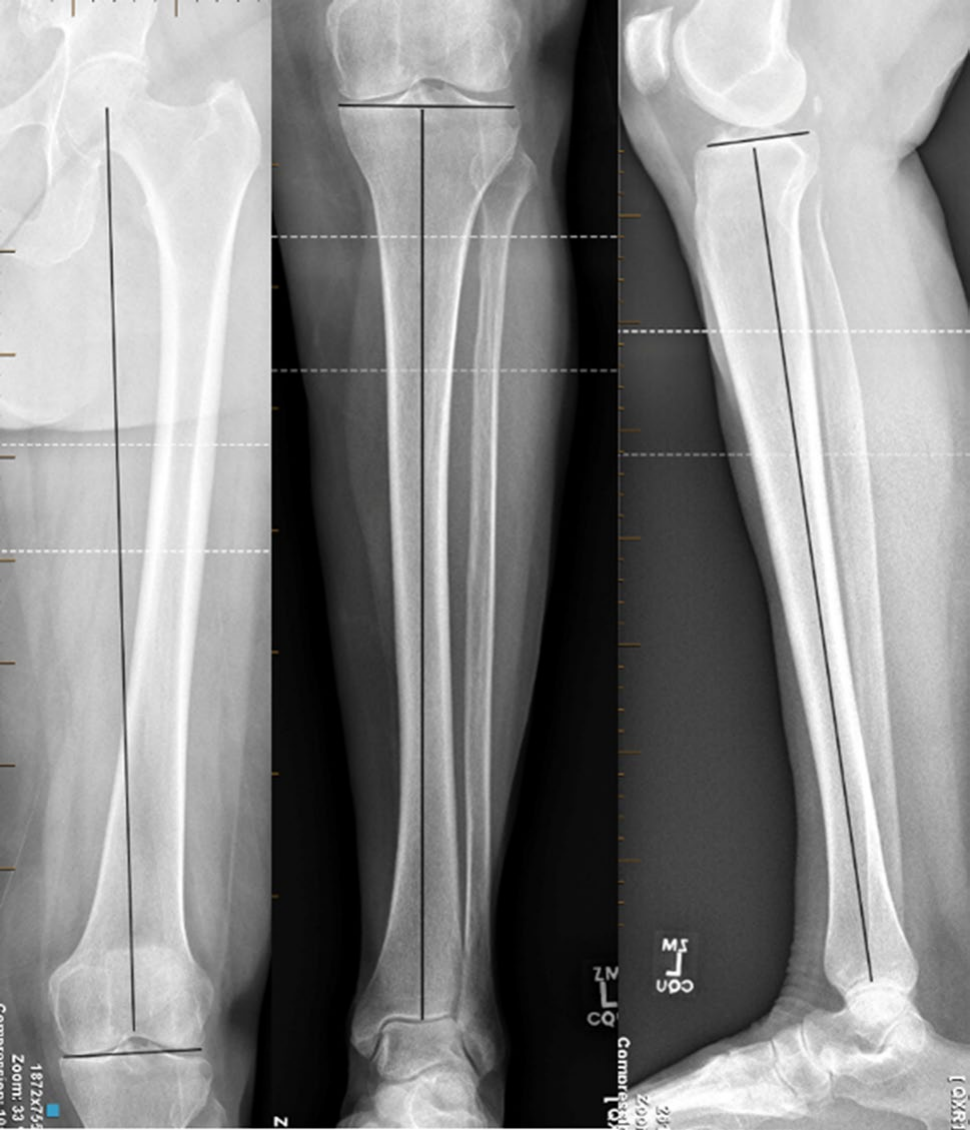
Radiographic parameters demonstrating measurement of the; mechanical Lateral Distal Femoral Angle (mLDFA), mechanical Proximal Tibial Angle (mPTA) and Posterior Tibial Slope (PTS)

**Fig. 2:**
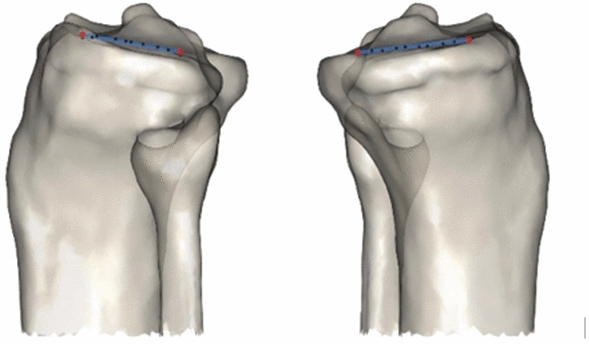
Differential Posterior Tibial Slopes; Lateral Posterior Tibial Slope (LPTS) and Medial Posterior Tibial Slope (MPTS)

Radiographic pre-operative assessment is considered mandatory. Appropriate radiographic parameters can be taken from either long limb standing radiographs, whole limb low dose standing computed tomography (CT) or standing EOS whole body scans. This series made exclusive use of long-limb standing radiographs and EOS scans. Hip-Knee-Ankle angle (HKA), mechanical Lateral-Distal-Femoral-Angle (mLDFA), mechanical proximal-tibial-angle (mPTA), and Posterior Tibial Slope (PTS) were all recorded (see Fig. 1). There was an excellent inter- and intra-rater reliability, both Kappa 0.9 (*p* < 0.001). Pre- and post-operative anatomical Tibio-Femoral (aTF) angle was recorded to permit KSS score alignment deductions where present. The posterior tibial slope is assessed both pre-operatively and checked intra-operatively once navigation points were registered to determine whether a significant (> 5 degrees) differential medial and lateral slope was present (see Fig. 2).

**Fig. 3:**
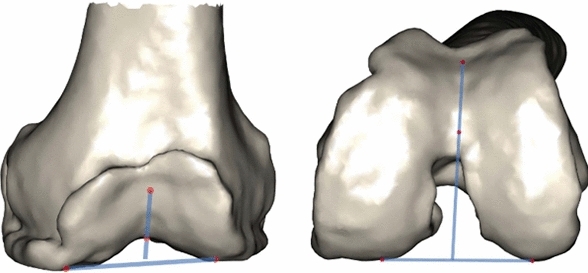
PFJ Radiographic parameters; Trochlea Angle to Distal Femoral Angle (TA-DFA) and Trochlea Angle to Posterior Condylar (TA-PCA)

When 3D scans such as CT and MRI were available, the angle formed between trochlear angle (TA) was measured against the distal femoral angle (DFA) to determine the TA-DFA and also against the posterior condylar axis (PCA) to determine the TA-PCA (see Fig. 3).

### Intra-operative data collection

An intraoperative data collection sheet was completed for all patients (Supplement 1). Anaesthesia type, implant manufacturer and polyethylene details, tourniquet times, resection size (in mm), resection angles (in degrees) and soft tissue releases (including ITB, Lateral release, LCL, MCL, PCL, Popliteus and Posterior capsule), were all recorded.

All patients underwent a standard medial parapatellar approach and removal of osteophytes before attaching navigation pins and CAS registration. It should be noted that the intra-articular navigation registration points are taken from the cartilage level, if present, as it is challenging to denude posterior cartilage at the time of initial registration. There may well be a difference between radiographic and intra-articular values due to variable cartilage thickness, and surgeons should consider this as a possible cause for minor mismatches between CAS data and recorded radiographic measures.

Two navigation systems were used (BrainLab®, Munich, Germany or Orthomap Precision®, Michigan, United States of America), measuring gaps to the nearest mm, depending on which TKR supplier was used with a previous quoted accuracy of 0.95, and inter- and inter-observer reliability of 0.95 and 0.96, respectively [3], or root-mean-square error of 0.13 [8]. Kinematic curves were recorded from the navigation unit at three time points. Initially, after navigation registration and removal of osteophytes, secondly with virtually planned resections and implants, and finally at case completion. At the time of kinematic curve data collection, stress was applied to the knee consistently through range from extension to flexion. This stress was always applied in the opposite direction of the most diseased compartment—valgus stress for varus knees and varus stress for valgus knees. Five types of curves were identified on the CAS kinematic curve screen. Curves were classified within in a three-degree window from their alignment, moving from full extension to maximal achievable flexion. The curves were namely 1. Straight, 2. Varus to Varus, 3. Varus to Valgus, 4. Valgus to Valgus, and 5. Valgus to Varus [20].

The medial and lateral tibio-femoral gaps were individually recorded (in mm) from the navigation unit in both extension and 90° flexion in both an unstressed and stressed condition giving eight values (medial extension unstressed, medial extension stressed, lateral extension unstressed, lateral extension stressed, medial flexion unstressed, medial flexion stressed, lateral flexion unstressed, and lateral extension stressed). These measurements were performed at two different time points. Firstly, following the removal of osteophytes and navigation registration and secondly, at the completion of the surgery. Unstressed gaps were measured by applying a gravity-led opening of each compartment in extension and 90° flexion. Stressed gaps were measured in the same four positions whilst a varus or valgus force was applied across the joint as previously described [7]. Finally, the laxity in each compartment was measured and recorded at the completion of the surgery. Laxity, in this instance, was defined as the delta between unstressed and stressed gaps in millimetres [20].

At the same time as the tibio-femoral gaps measurements, the HKA coronal alignment was measured in degrees in both extension and flexion. This was measured initially after navigation registration and removal of osteophytes in an unstressed state to gauge the arthritic alignment without osteophytes. This was then repeated in a stressed position to simulate the re-tensioning of the ligaments that occurs with the insertion of a prosthesis. The final HKA measurement was performed in an unstressed state in both flexion and extension at the completion of the case following definitive implant insertion.

Informed consent was obtained for each patient. Institutional Human Research and Ethics/Institutional Review Board (IRB) approval was granted by Mater Misericordiae Ltd, reference number: HREC/MML/67086.

## Statistical analysis

All data were analysed using the Statistical Package of Social Sciences (SPSS, Version 24). Continuous data were expressed as means and standard deviation. Number of subject data collected were quantified as “*n*”.

To determine differences between means (MD), for two independent data sets, paired t-test was used, and for one independent and one dependent data set, independent t-test was used. Statistical significance was defined as *p* < 0.05.

When comparing correlation, normality was assessed using Shapiro–Wilk, and skewness and kurtosis were assessed using z-values. When non-parametric correlation was assessed, Spearman’s correlation was utilised. SPSS Syntax MANOVA power analysis was performed and determined as per Cohen’s parameters, 1 − *ß*, > 0.8.

## Results

**Fig. 4:**
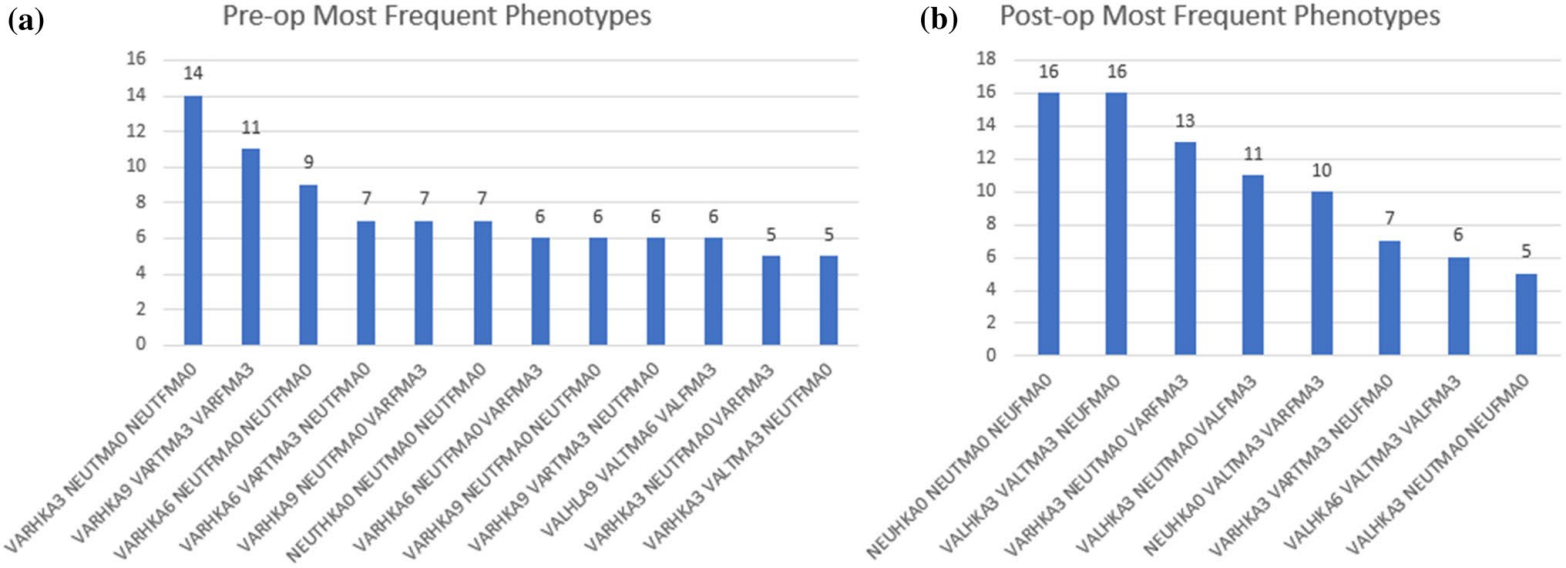
Pre- and post-operative functional phenotypes. **a** The twelve most frequent functional knee phenotypes measured pre-operatively. **b** The eight most frequent functional knee phenotypes measure post-operatively

Pre- and post-operative HKA, MTPA, LDFA and tibial slope are shown in Table 1. CPAK data are shown in Table 2. Hirschmann’s functional knee phenotype data pre- and post-operatively are shown in Fig. 4a/b. There were no significant differences in PROMs, or ROM, between the post-operative alignment classification groups, or post-op overall varus vs valgus HKA.

**Table 1:** Radiographic measurement of pre- and post-operative HKA, MTPA, LDFA and PTS

Pre- to post-operative measure	Mean	Range	Mean difference	*p* value
Pre-op HKA	− 3.8 ± 6.2	− 19 to 14	3.7 ± 4.8	< 0.001
Post-op HKA	0.1 ± 4	− 13 to 4		
Pre-op MTPA	− 2.9 ± 2.6	− 11 to 10	0.5 ± 2.7	0.03
Post op MTPA	− 2.4 ± 2.1	− 15 to 2		
Pre-op LDFA	2.5 ± 2.9	− 13 to 12	0.01 ± 2.6	N.S
Post op LDFA	2.5 ± 2.5	− 9 to 4		
Pre-op PTS	7.5 ± 3.3	− 5 to 8	2.1 ± 3.6	< 0.001
Post op PTS	5.4 ± 2.4	− 13 to 4

**Table 2:** CPAK

Pre-op CPAK	Frequency (*n*)	Percentage (%)	Post-op CPAK	Frequency (*n*)	Percentage (%)
1	34	20.4	1	20	12
2	63	37.9	2	59	35.5
3	27	16.3	3	30	18.07
4	11	6.6	4	12	7.2
5	21	12.7	5	35	21
6	8	4.8	6	5	3
7	2	1.2	7	2	1.2
8	0	0	8	0	0
9	0	0	9	0	0
CPAK Maintained Pre-op and post-op (%)					
Yes			No		
39.2			60.8

ROM arc pre-operatively was 118.6° (*n* = 165, SD ± 7.5, Min 97°, Max 136°), at 12-months; 118.6° (*n* = 161, SD ± 9.5, Min 70°, Max 138°) and 24-months 120.1° (*n* = 165, SD ± 8.2, Min 90, Max 135).

When reviewing curves (*n* = 159) the majority were straight pre-operatively, intra-operatively and post-operatively (Table 3). In terms of accuracy, 91.8% of patients had the same curve predicted (stress) and final.

**Table 3:** Kinematic curve prediction

	Straight		Varus to valgus		Varus to varus		Valgus to varus		Valgus to valgus	
	*N*	%	*N*	%	*N*	%	*N*	%	*N*	%
Pre-op	71	44.4	29	18.1	36	22.5	16	10	8	5
Predicted	101	63.9	16	10.1	31	19.6	8	5.1	2	1.3
Post-op	102	64.6	15	9.5	28	17.7	12	7.6	1	0.6

Intra-operative alignment data were collected in all subjects from the CAS navigation system (*n* = 165). Coronal alignment navigation data in extension demonstrated a significant change in the un-stressed pre-operative HKA versus the post-operative unstressed HKA with values of − 3.5° and − 1.5°, respectively (*p* < 0.0001). Power *ß* < 0.001, *p* < 0.001.

The CAS navigation measured pre-operative Stress HKA was compared with the post-operative CAS navigation measured unstressed HKA. This was compared in extension and 90 degrees of flexion. In extension, the pre-operative stressed coronal HKA versus post-operative unstressed HKA was − 1.4 and − 1.5, respectively (*p* = N.S). In 90 degrees of knee flexion the pre-operative stressed coronal HKA versus post-operative unstressed HKA were both 2.0, (*p* = N.S). Power *ß* < 0.001, *p* < 0.001.

**Fig. 5:**
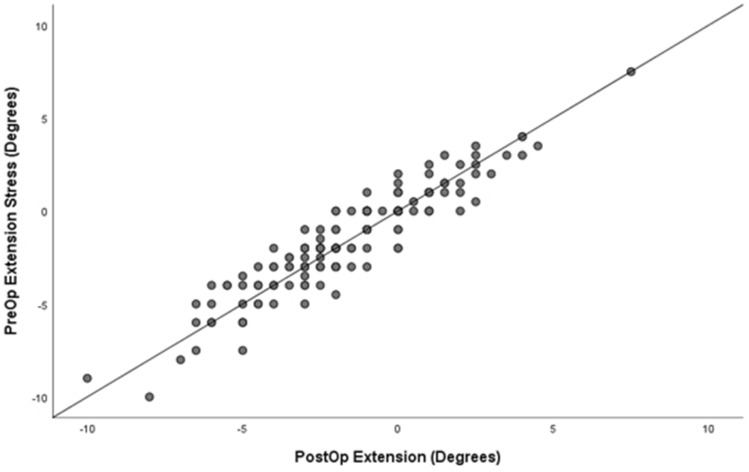
Scatterplot of Pre-op vs post-op alignment in extension

**Fig. 6:**
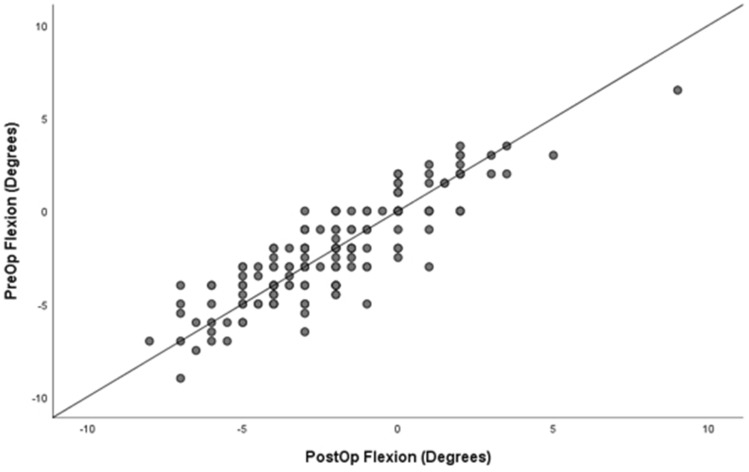
Scatterplot of Pre-op vs post-op alignment in flexion

When determining the validity of pre-operative stress value and correlation to postoperative unstressed alignment, there was a high correlation between pre-operative stress extension and post-op neutral extension alignment, *r* = 0.9, *p* < 0.001, and pre-operative stress flexion and post-operative flexion alignment, *r* = 1.0, *p* < 0.001. Accuracy was stratified to percentages within degrees of the pre-operative stress value and post-operative value in flexion and extension (Table 4). Scatterplot is demonstrated for extension in Fig. 5 and flexion in Fig. 6. Regression analysis is shown in Supplement 5 and 6 for extension and flexion, respectively.

**Table 4:** Degree of accuracy demonstrated on pre-operative stress alignment when compared to post-operative final alignment

	Pre- to post-extension		Pre- to post-flexion	
	*n*	%	*n*	%
0	51	30.9	43	27
1	87	51.8	67	42
2	26	15.5	47	30
3	1	0.6	1	0.6

Post-operative gaps and laxity are shown in Table 5, and the utilised soft tissue releases are summarised in Table 6.

**Table 5:** Pre- and post-operative gap and laxity measurements

Pre-op	Medial Gap	Extension	Mean	19.8 ± 1.34	Lateral Gap	Extension	Mean	20.5 ± 1.59
			Range	16–24			Range	16–25
		Flexion	Mean	18.8 ± 3.22		Flexion	Mean	20.5 ± 1.7
			Range	0–29			Range	16–29
Post op	Medial Gap	Extension	Mean	1.2 ± 0.9	Lateral Gap	Extension	Mean	2.0 ± 1.1
			Range	0–7			Range	0–7
		Flexion	Mean	1.5 ± 1		Flexion	Mean	4.2 ± 1.7
			Range	0–6			Range	0–8

*n* = 165. Power *ß* < 0.001, *p* < 0.001

**Table 6:** Soft tissue releases performed

Type of release	*n*	% Of releases	% Of total
Popliteus	1	4	0.6
PCL^a^	3	13	1.8
Lateral release^a^^b^	12	50	7.1
Posterior capsule^b^	7	29	4.2
ITB	1	4	0.6
			14.5

^a^One patient requiring PCL slide and lateral release

^b^One patient requiring posterior capsular and lateral releases

Three patients (1.8%) underwent re-operation for: extensor mechanism failure following significant trauma, intra-operative tibial fracture requiring stemmed revision prosthesis, and poly exchange to a larger size due to delayed presentation instability. This case of instability was determined by an excessive anterior drawer, in addition to patient complaints of instability, particularly when walking downstairs. (Supplement 7).

**Fig. 7:**
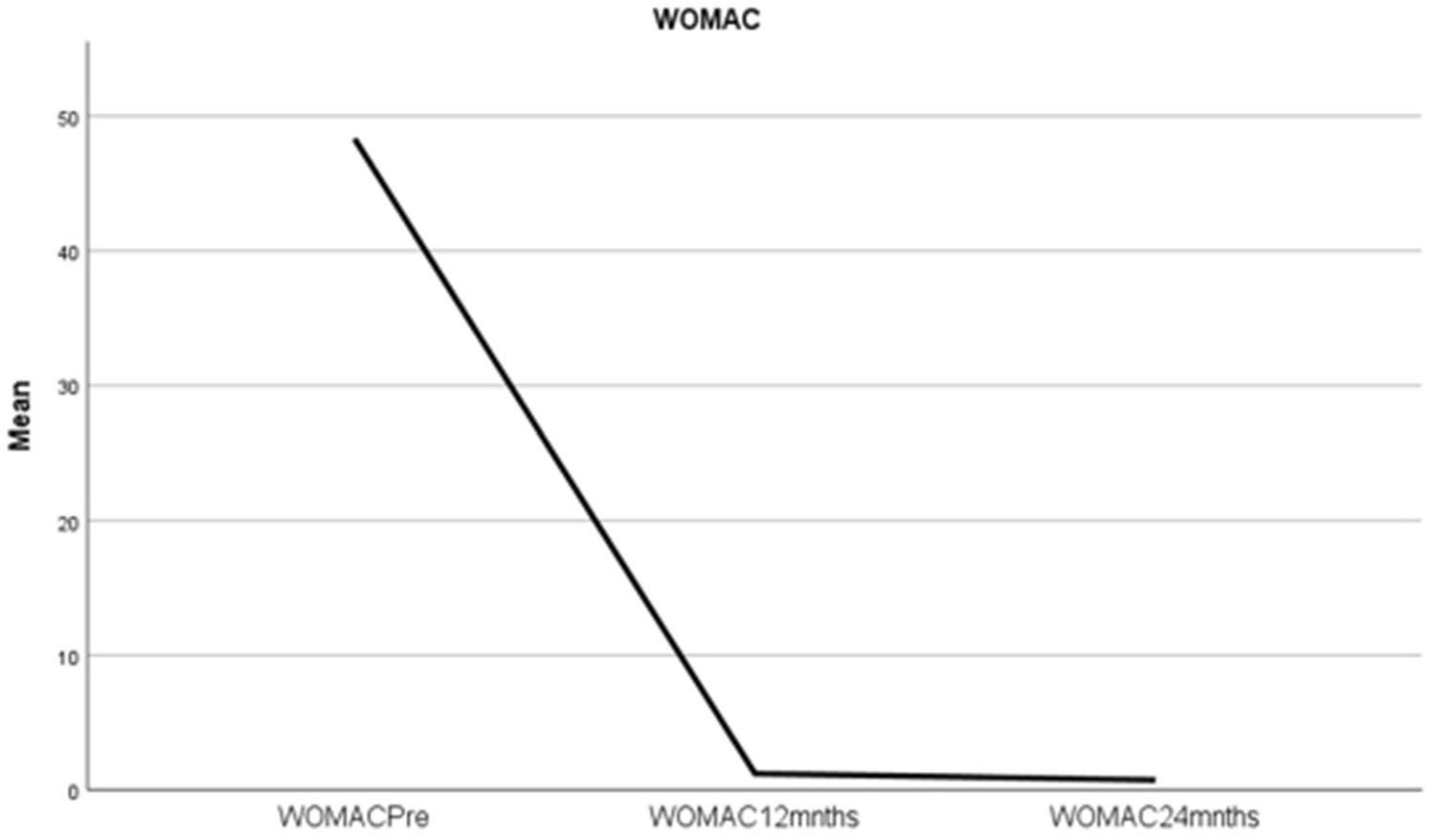
Mean WOMAC pre-op, at 12 months and at 24 months post-op. WOMAC pre-op, 12 months, 24 months, mean scores of 48.8 (SD ± 3.8), 1.4 (SD ± 3.9) and 1.2 (SD ± 5.2), power 24 months *ß* = 0.146, *p* = 0.003

**Fig. 8:**
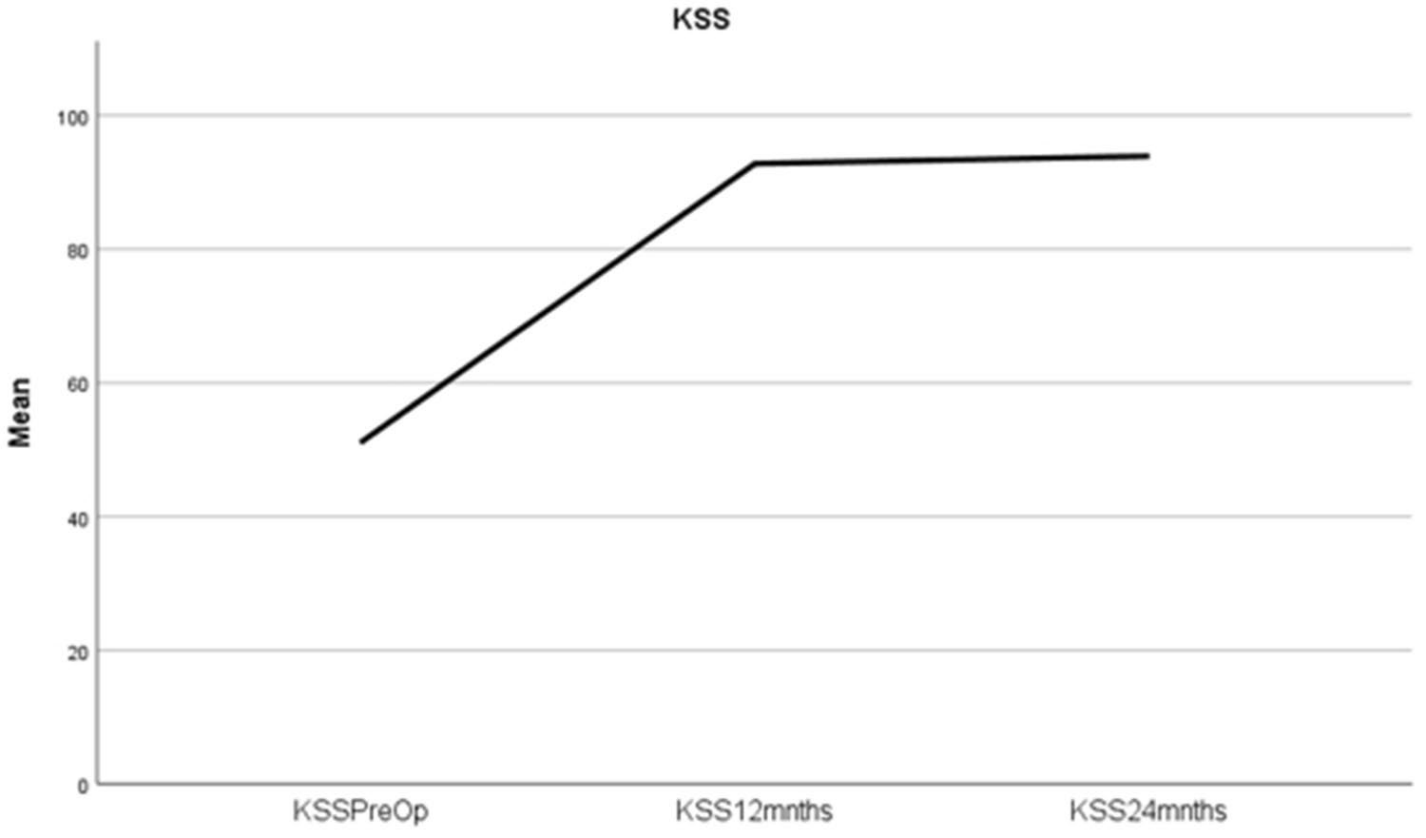
Mean KSS pre-op, at 12 months and at 24 months post-op and regards to KSS, 48.8 (SD ± 8.0), 92.5 (SD ± 7.2) and 93.7 (SD ± 5.8) respectively

WOMAC and KSS were collected at three time points (*n* = 165 at each). Results are shown in Figs. 7 and 8. Regarding KSS, 17 patients received alignment-based deductions**.** Implant specific comparison at 24 months demonstrated no significant difference; Stryker Triathlon, DePuy Attune cruciate-retaining and rotating platform, KSS were 94.5, 94.0 and 93.4, respectively. (see Supplement 3 for further results regarding implant specific data and comparison). Sub-analysis demonstrated there was no difference in WOMAC or ROM at any time point between those who received KSS alignment-based deductions and those who did not receive deductions.

## Discussion

Importantly, this is the first published results of a navigated FA TKA cohort and demonstrates that FA is reproducible, reliable, and a safe approach to knee arthroplasty without the need for robotics. The CAS navigation facilitates an accurate reproduction of functional alignment within the soft tissue boundaries, accurately reproducing preoperative stress values to realign the patient to the predicted pre-disease state. Thereby demonstrating functional alignment as a practical philosophy for those surgeons who do not have access to robotics.

There has been an increase in the use of assistive technology such as navigation and, more recently, robotics [10]. In 2021, 28.5% of TKR performed in Australia were using navigation, and 22.5% used robotics [1]. Although other reports of FA use robotic technology, this paper highlights that navigation may also be used to perform FA TKA successfully.

PROMs at 1 and 2 years demonstrated satisfaction scores for knees in both postoperative varus and valgus alignment, to be comparable to recently published data on robotic-arm assisted TKA; KSS 94.5 at 2 years [4]. Clarke et al. demonstrated a 99% medial extension gap < 2 mm with their robotic FA technique, which is comparable to this cohort’s mean of 1.2 ± 0.9 [5]. The results support the underlying rationale for CAS navigated FA being a reliable technique that does not require a robotic platform whilst still delivering acceptable PROMs and accuracy (see Supplement 1). Additionally, when reviewing the cases in this series adhering to the restricted functional alignment parameters set out by Oussedik et al. and considering them as “constrained FA” [21] to those considered “coronally unconstrained” (*n* = 30), there is no difference in PROMs or ROM.

When considering alignment classification, notably Hirschmann’s functional knee phenotypes and CPAK, as well as the radiographic parameters of HKA, MTPA and LDFA; all parameters trend towards the neutral alignment with a narrowed distribution curve. In particular, the postoperative variability of knee phenotypes reduced, indicating reliability in returning the knee a less disordered state more closely approximating the pre-disease state with measurably improved knee kinematics. Despite the various post-operative alignment classifications, there was no significant difference between the groups and their PROMs or ROM.

A key difference of FA over KA and other nMA’s is that FA not only attempts to realign the knee in the coronal plane in extension but also aims to do the same in flexion alignment, which typically receives less attention as it is more challenging to measure flexion alignment pre-operatively and post-operatively. Predicting and realigning the knee through its ROM curve allows the soft tissue to maintain appropriate tension, restore normal kinematic features such as medial pivot, and avoid flexion instability [26, 27]. Additionally, FA accepts that a compromise on implant alignment or position may be required to restore suitable kinematics. This philosophical concept of restoring soft tissue tension through range may be more important than the static knee coronal plane alignment in extension.

The soft tissue release rate was very low. Out of the 26 patients that had releases performed, 12 were lateral releases. Where pre-operative 3D imaging is available, operative planning involves assessment of the native trochlear angle in reference to the distal femoral angle and the posterior condylar line to determine the TA-DFA and TA-PCA respectively. These parameters can assist in identifying radiographic outliers and permit correlation to intra-operative mal-tracking. Studies have shown a wide variation in the trochlea anatomy that may not be addressable by the current techniques and implants [11]. Additionally, acting on pre-operative knowledge regarding differential tibial slopes may lead in some cases to deliberate femoral rotation to improve flexion kinematics that may inadvertently compromise the PFJ kinematics [28]. In cases where addressing PFJ mal-tracking with compensatory femoral component rotation was not desired due to the knock-on effects on flexion tibio-femoral kinematics, a lateral release was performed to address the compromised PFJ kinematics.

There are several limitations to this study. No comparison group makes it difficult to compare PROMs to established techniques outside of comparisons drawn from published data. There is no test–retest reliability measure for intraoperative measures. The paper has only a follow-up of two years, and therefore it is impossible to comment on the long-term implant survivorship. The laxity data rely on gravity-fed forces and therefore is not standardised. The long leg radiographs were performed at six weeks post-operative and although most patients had recovered full extension, some who were slow in the rehab might skew the final alignment assessment. Unlike other papers discussing FA, this iteration of FA is coronally unconstrained which may require consideration when drawing comparisons or discussing techniques.

## Conclusion

Navigated CAS FA TKA in this cohort proved to be a predictable, reliable, and reproducible technique with acceptable short-term revision rates and high PROMs. FA can account for individual patient bony morphology extremes and achieve desired gap and kinematic targets with low frequencies of soft tissue release required. Furthermore, the navigated FA results are comparable to those reported using robotic FA techniques.

## Supplementary Information

Below is the link to the electronic supplementary material.

Supplementary file1 (DOCX 12 KB)

Supplementary file2 (DOCX 31 KB)

Supplementary file3 (DOCX 28 KB)

Supplementary file4 (DOCX 20 KB)

Supplementary file5 (JPG 222 KB)

Supplementary file6 (JPG 25 KB)

Supplementary file7 (JPG 24 KB)

Supplementary file8 (DOCX 18 KB)

Supplementary file9 (JPG 25 KB)

Supplementary file10 (JPG 23 KB)

## Data Availability

The datasets generated during and/or analysed during the current study are available from the corresponding author on reasonable request.
