# Supplementary material for: Navigated functional alignment total knee arthroplasty achieves reliable, reproducible and accurate results with high patient satisfaction
Source: Knee Surg Sports Traumatol Arthrosc. 2023 Mar 14;31(9):3861–70. doi: 10.1007/s00167-023-07327-w (PMC10435654; doi:10.1007/s00167-023-07327-w)
Supplement: Supplementary file 1 — Supplementary file1 (DOCX 12 KB) [file 167_2023_7327_MOESM1_ESM.docx]

Supplement Legend

Supplement 1: An Expansion of the Purpose

Supplement 2: An Expansion of the Surgical Technique

Supplement 3: A Sub Analysis of the Different Prostheses

Supplement 4: Intra-operative data record for alignment measure

Supplement 5: Regression analysis in extension

Supplement 6: Regression analysis in flexion

Supplement 7: Details of patient requiring revision of poly

Supplement Figure 9: Mean WOMAC pre-op, at 12-months and at 24-months post-op by implant

Supplement Figure 10: Mean KSS pre-op, at 12-months and at 24-months post-op by implant
