# Supplementary material for: Navigated functional alignment total knee arthroplasty achieves reliable, reproducible and accurate results with high patient satisfaction
Source: Knee Surg Sports Traumatol Arthrosc. 2023 Mar 14;31(9):3861–70. doi: 10.1007/s00167-023-07327-w (PMC10435654; doi:10.1007/s00167-023-07327-w)
Supplement: Supplementary file 2 — Supplementary file2 (DOCX 31 KB) [file 167_2023_7327_MOESM2_ESM.docx]

**Supplement**

**Purpose – an Expansion**

The fundamental objective of FA is to reliably and accurately achieve a balanced total knee replacement, utilizing minimal soft tissue releases and standard TKA implants; that achieve knee kinematics that approximate those of the native/normative knee, as closely as possible. FA starts with an individualized pre-operative plan and proceeds by using bespoke CAS guided bony resections and repeated intra-operative measurements to guide subsequent resections, adapting them as necessary according to FA principles. The principal surgeon aims to reproduce the pre disease alignment is extension and flexion whilst balancing the knee within the native laxity envelope. This allows the tibia to rotate around the femur in the most soft tissue friendly alignment, as described in O’Callaghan et al[11].

The primary research hypothesis was that FA is a reliable technique when comparing the planned and executed FA Hip Knee Ankle alignment (HKA) within the experimental patient cohort. The secondary hypothesis was that predicted intra-operative kinematic curve would be predictive of the postoperative kinematic curve. A tertiary hypothesis was that FA could achieve a balanced knee with ligament sparing and gaps that could facilitate medial pivot. A final hypothesis was that FA is a safe technique with low revision rate and acceptable PROMs at 1 and 2 years.

This iteration of FA can be performed using a tibia-first or femur-first workflow although all cases in this series were performed using tibia-first technique. CAS is a mandatory requirement for FA TKA. FA dispenses with both the principles of mechanical 0° alignment and the goal of achieving symmetric rectangular flexion-extension gaps. Instead, it acknowledges the KA aim to restore the native pre-disease limb (constitutional) coronal alignment and joint obliquity. Like FA and derivatives, ligament releases are avoided. However, it deviates from KA in that it balances the ligaments in such a way that restores tissue tension in a stressed position. Native stress angles are used as a surrogate for constitutional pre disease alignment and identifying intra-operative alignment targets[4]. This process of “Stress Balancing” aims to replicate pre-arthritic physiologic medial and lateral tibio-femoral gap distances within the chosen constitutional alignment parameters at both 0° and 90° of knee flexion and restore a medial pivot mechanic to the knee[11]. This method has been validated by Tarasolli et al and is comparable to radiological arithmetic Hip-Knee-Ankle angle (aHKA)[10, 12]. It should be noted that when wear is global with increasing degrees of bone-loss then this process may be more difficult. The surgeon will have to estimate, determining the best fit alignment taking into consideration the amount of wear less damaged compartment. In the case of TKA for predominantly PFJ disease, no stress values are determined as there is no requirement to account for bone loss in the medial or lateral compartments. The initial alignment is the target alignment.

Over the past few years, there has been heightened interest in coronal knee alignment. MA, , is still considered to be the ‘gold standard’ [3, 6, 7]. The MA philosophy accepts the prosthesis will differ from the natural knee’s morphology but will be inserted in such a way as to achieve a biomechanically sound prosthetic knee. Adjusted Mechanical Alignment technique (aMA) is a recently evaluated modification of MA that permits under-correction of the coronal resection to match the constitutional varus/valgus deformity up to a restricted maximum of 3°[1, 2, 8, 9, 13]. However, constitutional limb alignment is often not neutral, and the joint line is infrequently perpendicular to the mechanical axis. Changing a knee from a constitutional alignment that is not 0°, as advocated by MA/aMA; can lead to soft tissue imbalance requiring releases and patient dissatisfaction[5] [14].

References

1. De Muylder J, Victor J, Cornu O, Kaminski L, Thienpont E (2015) Total knee arthroplasty in patients with substantial deformities using primary knee components. Knee Surg Sports Traumatol Arthrosc 23:3653-3659

2. Deep K, Eachempati KK, Apsingi S (2015) The dynamic nature of alignment and variations in normal knees. Bone Joint J 97-b:498-502

3. Diduch DR, Insall JN, Scott WN, Scuderi GR, Font-Rodriguez D (1997) Total knee replacement in young, active patients. Long-term follow-up and functional outcome. J Bone Joint Surg Am 79:575-582

4. Grant AL, Doma KD, Hazratwala K (2017) Determination of the accuracy of navigated kinematic unicompartmental knee arthroplasty: A 2-year follow-up. J Arthroplasty 32:1443-1452

5. Howell SM, Howell SJ, Kuznik KT, Cohen J, Hull ML (2013) Does a kinematically aligned total knee arthroplasty restore function without failure regardless of alignment category? Clin Orthop Relat Res 471:1000-1007

6. Insall JN, Binazzi R, Soudry M, Mestriner LA (1985) Total knee arthroplasty. Clin Orthop Relat Res 13-22

7. Insall JN, Hood RW, Flawn LB, Sullivan DJ (1983) The total condylar knee prosthesis in gonarthrosis. A five to nine-year follow-up of the first one hundred consecutive replacements. J Bone Joint Surg Am 65:619-628

8. Khatib Y, Xia A, Naylor JM, Harris IA, Sorial RM (2019) Different targets of mechanical alignment do not improve knee outcomes after TKA. Knee 26:1395-1402

9. Lee HJ, Lim JW, Lee DH, Kim DH, Park YB (2020) Slight under-correction using individualized intentional varus femoral cutting leads to favorable outcomes in patients with lateral femoral bowing and varus knee. Knee Surg Sports Traumatol Arthrosc 28:1579-1586

10. MacDessi SJ, Griffiths-Jones W, Harris IA, Bellemans J, Chen DB (2021) Coronal Plane Alignment of the Knee (CPAK) classification. Bone Joint J 103-b:329-337

11. O'Callaghan WB, Gouk C, Wilkinson MPR, Haztratwala K (2022) Computer-aided surgery-navigated, functional alignment total knee arthroplasty: A surgical technique. Arthroplast Today 14:121-127

12. Tarassoli P, Wood JA, Chen DB, Griffiths-Jones W, Bellemans J, MacDessi SJ (2022) Arithmetic hip-knee-ankle angle and stressed hip-knee-ankle angle: equivalent methods for estimating constitutional lower limb alignment in kinematically aligned total knee arthroplasty. Knee Surg Sports Traumatol Arthrosc;10.1007/s00167-022-07038-8

13. Vanlommel L, Vanlommel J, Claes S, Bellemans J (2013) Slight undercorrection following total knee arthroplasty results in superior clinical outcomes in varus knees. Knee Surg Sports Traumatol Arthrosc 21:2325-2330

14. Vigdorchik JM, Wakelin EA, Koenig JA, Ponder CE, Plaskos C, DeClaire JH, et al. (2022) Impact of component alignment and soft tissue release on 2-year outcomes in total knee arthroplasty. J Arthroplasty;10.1016/j.arth.2022.04.042
