# Supplementary material for: Navigated functional alignment total knee arthroplasty achieves reliable, reproducible and accurate results with high patient satisfaction
Source: Knee Surg Sports Traumatol Arthrosc. 2023 Mar 14;31(9):3861–70. doi: 10.1007/s00167-023-07327-w (PMC10435654; doi:10.1007/s00167-023-07327-w)
Supplement: Supplementary file 3 — Supplementary file3 (DOCX 28 KB) [file 167_2023_7327_MOESM3_ESM.docx]

**Supplement**

**Explanation of FA Rationale and Surgical Technique:**

To achieve the desired stress balance parameters and soft tissue driven medial pivot, FA philosophy considers the sagittal and axial planes and treats the implant as freely mobile 3D object to be manipulated within the soft tissue envelope. In contrast to KA, this iteration of FA acknowledges that there is considerable variation in differential plateau posterior tibial slope (PTS) and patello-femoral joint (PFJ) morphologies[3]. Additionally, there are geometric constraints imposed by off the shelf implants and this technique can account for mismatches incurred by anatomical morphology variations by manipulating the prosthesis position beyond that prescribed by KA positioning to achieve a compromise that results in “best fit” kinematics[5].

FA relies on the advanced abilities afforded by certain navigation or robotic systems to not only achieve accurate resections but also to specifically perform “virtual” cuts to test knee balance intra-operatively prior to resections being made. Using the prosthesis planning screens, the surgeon is able to check knee alignment and perform varus/valgus stress manoeuvres on the knee, then adjust the virtual prosthesis position to achieve the desired soft tissue balance prior to actual bony resections. As bone is progressively resected, subsequent bony resections can also be virtually performed, and the planned prosthesis position is fine-tuned to optimize knee balance in a controlled and reproducible way. The result of each case should be measurable with objective alignment and kinematic data stored on the navigation or robotic computer unit. The underpinning ambition for this FA technique was to develop a bespoke method of performing knee arthroplasty that respects individual anatomy but can be executed in a reliable and reproducible way that is easy for surgeons to learn without any significant intra-operative time penalty intraoperatively or a requirement for non-standard instruments or implants.

Given the newness of the terminology of FA in the literature, to avoid confusion it should be clarified that this iteration of FA is performed with navigation, is coronally un-restricted (differing from other FA reports), is sagittally restricted to a maximum PTS and/or combined femoral flexion and PTS; and aims to avoid soft tissue releases unless absolutely essential with a marked preference for bony re-cuts to achieve soft tissue balance[1, 2, 4-7].

Colour Coded Tables – FA Algorithm

|  | **Isolated Asymmetric Medio-lateral Tightness/Laxity** | | | |
| --- | --- | --- | --- | --- |
|  | **Extension** | | **Flexion** | |
|  | **Medial** | **Lateral** | **Medial** | **Lateral** |
| **CAS Data Read Out & Clinical Assessment** | 1. TIGHT Medial Extension Gap: Relative Valgus to Predicted stress HKA. <1mm stress gap medially. Difficulty inserting poly/spacer block due to medial tightness | 1. TIGHT Lateral Extension Gap: Relative Varus to predicted stress HKA. <1mm stress gap laterally. Difficulty inserting poly/spacer block due to lateral tightness | 1. TIGHT Medial Flexion Gap: Relative Valgus to Predicted stress HKA. <1mm stress gap medially. Poly spits out medially in flexion/difficulty inserting spacer block | 1. TIGHT Lateral Flexion Gap: Relative Varus to predicted stress HKA. <1mm stress gap laterally |
|  | 3. LOOSE Medial Extension Gap: Relative Varus to predicted stress HKA. >2mm medial stress gap. Alignment corrected with valgus stress force | 3. LOOSE Lateral Extension Gap: Relative Valgus to Predicted stress HKA. >3mm lateral stress gap. Alignment corrected with varus stress force | 3. LOOSE Medial Flexion Gap: Relative Varus to predicted stress HKA. >2mm medial stress gap. Alignment corrected with varus stress force | 3. LOOSE Lateral Flexion Gap: Relative Valgus to Predicted stress HKA. >5mm lateral stress gap. Alignment corrected with valgus stress force |
| **Surgical Solution** | 1. TIGHT Medial Extension Gap: Resect additional 1° = 1mm resection off the medial femoral condyle | 1. TIGHT Lateral Extension Gap: Resect additional 1° = 1mm resection off the lateral femoral condyle | 1. TIGHT Isolated Medial Flexion Gap: Externally rotate the femur pivoting off the lateral condyle - resect more bone off the medial posterior condyle | 1. TIGHT Isolated Lateral Flexion Gap: Internally rotate the femur pivoting off the medial condyle - resect more bone off the lateral posterior condyle |
|  | 3. LOOSE Medial Extension Gap: Under-resect by 1° = 1mm less resection off the medial femoral condyle | 3. LOOSE Isolated Lateral Extension Gap: Under-resect by 1° = 1mm less resection of the lateral femoral condyle | 3. LOOSE Isolated Medial Flexion Gap: Internally Rotate (IR) the femur pivoting off the lateral femoral condyle i.e., resect 1mm less bone off the medial posterior condyle but keep lateral condylar resection unchanged | 3. LOOSE Isolated Lateral Flexion Gap: Externally Rotate (ER) the femur pivoting off the medial femoral condyle i.e., resect 1mm less bone off the lateral femoral condyle but keep medial condylar resection unchanged |

|  | **Symmetric Tightness/Laxity in Flexion-Extension Gaps** | **Asymmetric Tightness/Laxity in Flexion-Extension Gaps** |  |
| --- | --- | --- | --- |
|  |  |  |  |
|  |  |  |  |
| **CAS Data read out** | 1. TIGHT: FFD>5° or <1mm stress gaps in extension/flexion | 1. TIGHT Extension Gap: FFD>5° but appropriate flexion stress gap |  |
|  | 2. LOOSE: Hyper Extension >5° of native with >1+mm stress gaps outside of desired range | 2. TIGHT Flexion Gap: Appropriate extension but tight flexion stress gap |  |
|  |  | 3. LOOSE Extension Gap: Hyper Extension >5° of native but appropriate flexion gap |  |
|  |  | 4. LOOSE Flexion Gap: Appropriate extension but loose flexion gap |  |
| **Clinical Assessment** | 1. TIGHT: Unable to fully extend and poly squeaks and/or spits out in flexion | 1. TIGHT Extension Gap: Unable to fully extend but good flexion balance |  |
|  | 2. LOOSE: Hyper Extending knee with excessive medial and lateral gaps/laxity | 2. TIGHT Flexion Gap: Can fully extend but poly squeaks and/or spits out in flexion |  |
|  |  | 3. LOOSE Extension Gap: Hyper Extending knee with excessive extension medial and lateral gaps but good flexion balance |  |
|  |  | 4. LOOSE Flexion Gap: Can fully extend but flexion gaps are excessive |  |
| **Response/**  **Solution** | 1. TIGHT: Resect more of tibia | 1. TIGHT Extension Gap: Recut femur migrating proximally OR recut tibia more distally with less posterior slope |  |
|  | 2. LOOSE: Increase polyethylene thickness | 2. TIGHT Flexion Gap: Recut tibia with more posterior slope |  |
|  |  | 3. LOOSE Extension Gap: Recut tibia with more posterior slope AND increase poly size |  |
|  |  | 4. LOOSE Flexion Gap: Recut tibia with less posterior slope AND increase poly size |  |

References

1. Chang JS, Kayani B, Wallace C, Haddad FS (2021) Functional alignment achieves soft-tissue balance in total knee arthroplasty as measured with quantitative sensor-guided technology. Bone Joint J 103-b:507-514

2. Clark GW, Esposito CI, Wood D (2022) Individualized Functional Knee Alignment in Total Knee Arthroplasty: A Robotic-assisted Technique. Techniques in Orthopaedics;10.1097/bto.0000000000000567

3. Hazratwala K, O'Callaghan WB, Dhariwal S, Wilkinson MPR (2021) Wide variation in tibial slopes and trochlear angles in the arthritic knee: a CT evaluation of 4116 pre-operative knees. Knee Surg Sports Traumatol Arthrosc;10.1007/s00167-021-06725-2

4. Kayani B, Konan S, Tahmassebi J, Oussedik S, Moriarty PD, Haddad FS (2020) A prospective double-blinded randomised control trial comparing robotic arm-assisted functionally aligned total knee arthroplasty versus robotic arm-assisted mechanically aligned total knee arthroplasty. Trials 21:194

5. O'Callaghan WB, Gouk C, Wilkinson MPR, Haztratwala K (2022) Computer-aided surgery-navigated, functional alignment total knee arthroplasty: A surgical technique. Arthroplast Today 14:121-127

6. Oussedik S, Abdel MP, Victor J, Pagnano MW, Haddad FS (2020) Alignment in total knee arthroplasty. Bone Joint J 102-b:276-279

7. Steer R, Tippett B, Khan RN, Collopy D, Clark G (2021) A prospective randomised control trial comparing functional with mechanical axis alignment in total knee arthroplasty: study protocol for an investigator initiated trial. Trials 22:523
