# Supplementary material for: Navigated functional alignment total knee arthroplasty achieves reliable, reproducible and accurate results with high patient satisfaction
Source: Knee Surg Sports Traumatol Arthrosc. 2023 Mar 14;31(9):3861–70. doi: 10.1007/s00167-023-07327-w (PMC10435654; doi:10.1007/s00167-023-07327-w)
Supplement: Supplementary file 4 — Supplementary file4 (DOCX 20 KB) [file 167_2023_7327_MOESM4_ESM.docx]

**Supplement – Prosthetic Specific Subanalysis**

**Results**

**Prosthetic Specific Results**

Stryker Triathlon (TM etc.) was used in 26 (16%) and DePuy Attune Cruciate Retaining (CR) was used in 52 (31%). DePuy Attune Rotating Platform (RP) in 87 (52%). Polyethylene size inserts; in 96.4% the smallest poly available was utilised; broken down into each implant; Stryker: 9mm; n=23 (95.8%), 11mm; n=1 (4.2%). Attune: 5mm; n=52 (100%). Attune RP: 5mm; n=84 (94.4%), 6mm; n=4 (4.5%), 7mm; n=1 (1.1%).

**Prosthetic specific**

Supplement Figure 8 outlines the WOMAC, and Supplement Figure 9 outlines the KSS scores at 3 time periods; pre-operative, 12-months and 24-months post operative, by individual prosthesis type.

PROMS are subdivided into the prosthesis type (Table 1). There was no statistical difference between the post op outcome scores between the 3 major types of prosthesis used, however when comparing groups pre-op scores there was a statistically significant difference between groups when comparing Stryker Triathalon and Attune RP in both WOMAC and KSS; with Stryker Triathalon having a better mean pre-op WOMAC of 47.9, compared with Attune RP of 49.3 (p=N.S) and a better KSS of 53.5 compared to 46.3 respectively (p=<0.001), and when comparing Attune CR and RP KSS, Attune CR having a better mean score of 51.0 and Attune RP a worse mean score of 46.3 (p<0.001).

Comparison of the PROMs for the Stryker Triathalon and Attune CR were: WOMAC pre-op mean difference (MD); 0.3 (p=N.S), 12-months MD; 0.5 (p=N.S), 24-months MD; 0.01 (p=N.S), KSS pre-op MD 2.4 (p=N.S), 12-months MD 1.5 (p=N.S), 24-months 0.9 (p=N.S).

Comparison of the PROMs for the Stryker Triathalon and Attune RP were: WOMAC pre-op MD; 1.4 (p=0.01), 12-months MD; 0.3 (p=N.S), 24-months MD; 1.0 (p=N.S), KSS pre-op MD 7.2 (p<0.001), 12-months MD; 0.7 (p=N.S), 24-months MD; 0.8 (p=N.S)

Comparison of the PROMs for the Attune CR and Attune RP were: WOMAC pre-op MD; 1.1 (p=N.S), 12-month MD; 0.2 (p=N.S), 24-month MD; 0.9 (p=N.S). KSS pre-op MD; 4.7 (p<0.001), 12-months MD; 0.9 (p=N.S), 24-months MD: 0.4 (p=N.S).

Supplement Table 1 outlines the raw mean outcome scores for the 3 different prostheses.

**Discussion – An Expansion – Prosthetic Specific**

This study utilized 3 different prostheses from 2 manufacturers and the technique has proven agnostic to these implant choices and designs. Though there was a significant difference recorded in the preoperative PROMS for the Triathalon CR vs Attune RP and the Attune RP vs Attune CR; the postoperative PROMS were similar at the 1yr and 2yr assessment. Additionally, the PROMS results are comparable to multiple studies published looking at outcomes of knee replacement[1].

**Conclusion – An Expansion – Prosthetic Specific**

FA was implant agnostic with regard to the prostheses used in this series with no differences in outcome regardless of the prostheses used and worked within implant design limitations

|  | Mean WOMAC (SD) | | | Mean KSS (SD) | | |
| --- | --- | --- | --- | --- | --- | --- |
|  | Pre-op | 12-month | 24-months | Pre-op | 12-months | 24- months |
| Stryker | 47.9 (2.0) | 1.7  (4.6) | 0.7  (3.1) | 53.5  (5.6) | 91.6 (10.709) | 94.5  (5.0) |
| Attune CR | 48.2 (5.2) | 1.3  (3.3) | 0.7  (1.7) | 51.0  (6.2) | 93.2  (5.8) | 94.0  (4.2) |
| Attune RP | 49.3 (3.2) | 1.4  (4.1) | 0.8  (2.8) | 46.3 (8.6) | 92.3  (6.8) | 93.4  (6.8) |

Supplement Table 1: Mean PROMs by Implant over time

References

1. McEwen PJ, Dlaska CE, Jovanovic IA, Doma K, Brandon BJ (2020) Computer-assisted kinematic and mechanical axis total knee arthroplasty: A prospective randomized controlled trial of bilateral simultaneous surgery. J Arthroplasty 35:443-450
