# Supplementary figures and images for: Navigated functional alignment total knee arthroplasty achieves reliable, reproducible and accurate results with high patient satisfaction
Source: Knee Surg Sports Traumatol Arthrosc. 2023 Mar 14;31(9):3861–70. doi: 10.1007/s00167-023-07327-w (PMC10435654; doi:10.1007/s00167-023-07327-w)

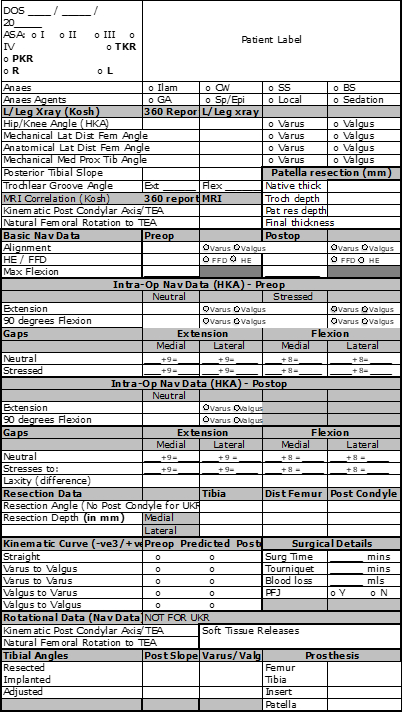

Supplement: Supplementary file 5 — Supplementary file5 (JPG 222 KB) [file 167_2023_7327_MOESM5_ESM.jpg]

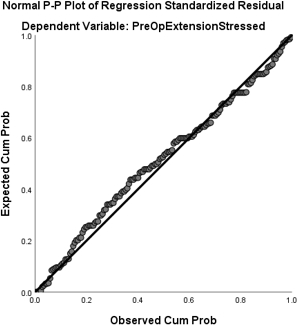

Supplement: Supplementary file 6 — Supplementary file6 (JPG 25 KB) [file 167_2023_7327_MOESM6_ESM.jpg]

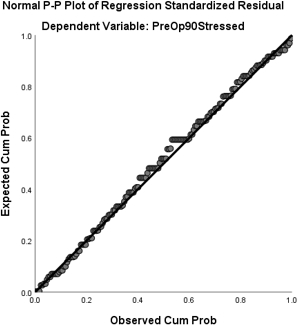

Supplement: Supplementary file 7 — Supplementary file7 (JPG 24 KB) [file 167_2023_7327_MOESM7_ESM.jpg]

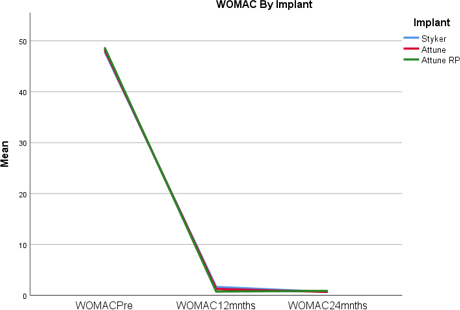

Supplement: Supplementary file 9 — Supplementary file9 (JPG 25 KB) [file 167_2023_7327_MOESM9_ESM.jpg]

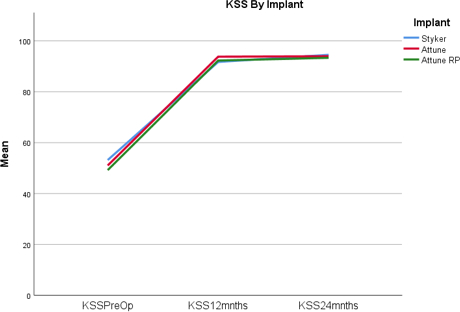

Supplement: Supplementary file 10 — Supplementary file10 (JPG 23 KB) [file 167_2023_7327_MOESM10_ESM.jpg]
