# Supplementary material for: Navigated functional alignment total knee arthroplasty achieves reliable, reproducible and accurate results with high patient satisfaction
Source: Knee Surg Sports Traumatol Arthrosc. 2023 Mar 14;31(9):3861–70. doi: 10.1007/s00167-023-07327-w (PMC10435654; doi:10.1007/s00167-023-07327-w)
Supplement: Supplementary file 8 — Supplementary file8 (DOCX 18 KB) [file 167_2023_7327_MOESM8_ESM.docx]

**Supplement 7 – Laxity Outliers & 1x Revision for Instability:**

**Laxity outliers:**

**There are two patients with high postoperative medial laxity numbers:**

Patient 32 - medial laxity of 5 degrees in extension

Patient 35 - medial laxity of 7 degrees in extension

All other readings are <4 degrees

**Lateral laxity:**

Patient 71 - lateral laxity of 8 in extension

Many patients have laxity of 7 in extension

In all 3 patients the BMI was noted to be >31. This can make accurate laxity assessment more difficult and increases the chance of tracker movement at the end of the case due to the increased requirement to handle the limb. None of the patients with increased postoperative laxity measurements have required revision

**Revision for Instability:**

**Patient 88 required right knee revision for instability:**

- 68 male (at time of surgery).
- Active field hockey player at master’s level state competition.
- Pre op WOMAC 53/100 and HSS 48/100

**Bilateral TKA was performed on 29/11/2018 with the following intra-operative details:**

Post-op Gaps and laxity

|  |  | Medial Extension | Lateral Extension | Medial Flexion | Lateral Flexion |
| --- | --- | --- | --- | --- | --- |
| Right | Gap | 21 | 21 | 19 | 20.5 |
|  | Laxity | 0 | 1 | 0.5 | 6 |
|  |  |  |  |  |  |
| Left | Gap | 22 | 22 | 19 | 19.5 |
|  | Laxity | 1 | 4 | 2 | 4 |

Navigation data following definitive implant insertion:

- Right TKA 3° FFD to 123° flexion
- Left TKA 0-120° flexion
- Final post op gaps, laxity and ROM of the definitive implants were within acceptable range.

**12 months postoperative progress:**

- 6/100 for WOMAC and 76/100 for HSS PROMs
- Patient had returned to playing hockey one year postoperatively.

**18 months postoperative progress:**

- Patient started developing effusion post exercise and pain whilst playing hockey.
- Patient stopped playing hockey but the effusion did not settle down.
- Clinically it appeared that the patient had lateral ITB pain as well as AP instability and was now hyperextending despite the intra-op nav data reporting that the right TKA was in 3° FFD at end of operation
- Infection was excluded and a trial of ITB stretching exercises was undertaken with physiotherapist oversight without benefit.
- An arthroscopic ITB release was performed.

**24 months postoperative progress:**

- The patient’s symptoms worsened after the arthroscopy and ITB release
- A subsequent minor revision was performed 2 years postoperatively to increase the polyethylene thickness by 2mm.
- This addressed the hyperextension and relieved the patient’s symptoms, allowing them to return to playing field hockey
